# Acute organ injury and long-term sequelae of severe pneumococcal infections

**DOI:** 10.1186/s41479-023-00110-y

**Published:** 2023-03-05

**Authors:** Katherine L. Kruckow, Kevin Zhao, Dawn M.E. Bowdish, Carlos J. Orihuela

**Affiliations:** 1 Department of Microbiology, University of Alabama at Birmingham, Birmingham, AL USA; 2 McMaster Immunology Research Centre and the Firestone Institute for Respiratory Health, McMaster University, Hamilton, Canada

**Keywords:** *Streptococcus pneumoniae*, Pneumonia, Invasive pneumococcal disease (IPD), Organ injury, Inflammation, Pneumolysin, Sequelae, Adverse cardiac events, Acute kidney injury, Mortality

## Abstract

*Streptococcus pneumoniae* (*Spn*) is a major public health problem, as it is a main cause of otitis media, community-acquired pneumonia, bacteremia, sepsis, and meningitis. Acute episodes of pneumococcal disease have been demonstrated to cause organ damage with lingering negative consequences. Cytotoxic products released by the bacterium, biomechanical and physiological stress resulting from infection, and the corresponding inflammatory response together contribute to organ damage accrued during infection. The collective result of this damage can be acutely life-threatening, but among survivors, it also contributes to the long-lasting sequelae of pneumococcal disease. These include the development of new morbidities or exacerbation of pre-existing conditions such as COPD, heart disease, and neurological impairments. Currently, pneumonia is ranked as the 9^th^ leading cause of death, but this estimate only considers short-term mortality and likely underestimates the true long-term impact of disease. Herein, we review the data that indicates damage incurred during acute pneumococcal infection can result in long-term sequelae which reduces quality of life and life expectancy among pneumococcal disease survivors.

## Introduction

*Streptococcus pneumoniae* (*Spn*) is the most common etiological agent of community-acquired pneumonia (CAP), bacteremia, and sepsis; in low or low-middle-income countries with no immunization schedule it is also the leading cause of meningitis [1–3]. Accordingly, the pneumococcus is the leading cause of infectious death in both children and older adults [4–8]. *Spn* is also a common cause of non-life-threatening infections including sinusitis, otitis media, and bronchitis which are associated with major personal and socio-economic costs [9–13]. This opportunistic, Gram-positive bacterium most often resides asymptomatically in the nasopharynx but can be aspirated into the lungs to cause pneumonia [8]. From the airway, the pneumococcus can gain access to the bloodstream and spread to other sites, where it can cause disseminated organ damage [14–16]. Young children with underdeveloped immune systems, those with immunodeficiencies, adults over the age of 65 with multiple comorbidities, and individuals experiencing or who have recently experienced a respiratory viral infection have increased susceptibility to both becoming infected with and dying from pneumococcal disease [5, 16–18].

It is worth noting that in most pneumococcal pneumonia cases, i.e., those that do not require hospitalization, there is typically no long-term negative effect. Among those with severe pneumonia, and despite the dense consolidation that can occur, the lungs radiologically return to normal by 2–4 months and most patients recover fully from all residual symptoms within 6 to 18 months [19, 20]. Yet, pneumococcal infections can exacerbate underlying pulmonary diseases, such as COPD. They can also cause a tremendous amount of system-wide damage. This damage is attributable to cytotoxic factors released by the bacterium, physiological stress put on the body during the infection, and an overzealous inflammatory response [21–36]. Strikingly, individuals previously hospitalized for invasive pneumococcal disease typically die sooner than would be expected based on census data; with nearly a decade of mean years of life lost being [37]. This reduction in life expectancy can be attributed to systemic organ damage during the infection [37, 38].

Sir William Osler was the first to describe the triad of pneumonia, endocarditis, and meningitis, and observed the presence of ‘micrococci’ in the affected tissues and blood in histological specimens at post-mortem examination; these ‘micrococci’ were later classified by Robert Austrian as *S. pneumoniae,* which is how this triad of disease developed the eponym of Austrian syndrome [39–41]. Austrian’s work highlighted the systemic consequences of pneumococcal pneumonia and that severe damage occurred when bacteria gained access to the bloodstream and invaded tissues [39, 41]. In accordance with this, *Spn* is well documented to cause acute damage to the middle ear, lungs, heart, and kidneys [9, 42–47]. Should the bacterium gain access to the central nervous system (CNS), there are especially devastating consequences to the affected individual with a case fatality rate of 30% in developed countries and up to 50% in lower-income countries [1, 7, 21, 22, 48–52]. As is discussed below, the damages incurred during pneumococcal infections persist well after the resolution of the acute infection and increase the risk for morbidity and mortality in convalescence. Many reviews on pathogenesis and the immunological events that take place during pneumococcal infection are available, but few have focused on the long-term consequences of this disease. This review examines the role the pneumococcus plays in tissue and organ damage during infection and the long-term sequelae associated with this damage. Sir William Osler famously called *Spn* “the Captain of all the men of death”; herein, we state that “the Captain lingers”.

## Acute pneumonia and invasive disease are highly inflammatory episode

Pneumococci that are aspirated into the lungs first encounter the mucous layer, which serves as a protective mechanical barrier for the mucosal epithelium, and sentinel alveolar macrophages (AMs). These early defenses serve to prevent the dissemination of pneumococci and initiate innate immune signaling and host cell activation [53, 54]. The mucous layer lining the epithelium traps bacteria in its sticky matrix and contains both microbicidal and microbiostatic products such as defensins, lactoferrin, lysozyme, matrix metalloproteinases (MMPs), antibodies, surfactant, complement proteins, and C-reactive protein (CRP) [54–61]. Upon contact with *Spn*, secretory IgA and complement proteins, C3 and C5a, aggregate and opsonize *Spn*, respectively, facilitating physical clearance and phagocytosis of the bacteria [56, 62]. Lysozyme, lactoferrin, b-defensin peptides, and LL-37 are directly bactericidal. However, the death of pneumococci, either by the host or the autolysis of itself, not only results in bacterial clearance but also in the release of inflammatory agents such as the pore-forming toxin, pneumolysin, and bacterial cell wall particles that intensify local inflammation [24–26, 54, 61, 63–70].

Pathogen recognition receptors (PRRs), including but not limited to Toll-like receptors (TLRs) and NOD-like receptors (NLRs), on both somatic airway epithelial cells and innate immune cells, particularly AMs, recognize pathogen-associated molecular patterns (PAMPs) and damage-associated molecular patterns (DAMPs) present during infection, triggering an inflammatory response to clear the pneumococcus [64]. TLRs-1, 2, 6, and 9 play a critical role in initiating the innate immune response, and their activation of B and T cells also promotes an adaptive immune response [67–69]. TLR-1/2 and TLR2/6 heterodimers expressed on host cells recognize teichoic acids and lipoproteins that are part of *Spn’*s cell wall [67–69, 71]. TLR9 detects DNA with unmethylated CpG motifs present in bacterial but not mammalian DNA [72]. TLR-4 is activated by complement and has been reported to detect *Spn’s* pneumolysin, but conflicting results have been published [64, 68, 73].

The binding of TLRs to their ligands engages adaptor molecules that transduce signals to activate proinflammatory signaling cascades including mitogen-activated protein kinases (MAPKs) and nuclear factor kappa B (NF-κB) [68, 71, 73, 74]. Upon TLR activation, sentinel AM in the airway rapidly signal to epithelial cells locally with cytokines and recruit neutrophils and monocytes via chemokines including tumor necrosis factor (TNF), interleukin (IL)-1β, IL-6, IL-8, CXCL1, CXCL2, and CCL2 [75–79]. Considerable evidence shows that *Spn* can invade host cells at low frequency, in which case the PAMPs from internalized *Spn* can interact with another class of PRRs, NLRs, that are found in the cytoplasm of host cells. NLRs have been shown to recognize peptidoglycan motifs via NOD1 and NOD2 which activate signaling cascades and NLRP3 that serves as a component of the inflammasome [80–83]. Upon NLRP3 detection of PAMPs or DAMPs, NLRP3 oligomerizes with other components to form inflammasomes, which in turn cleave the pro-forms of IL-1β and IL-18 to be released [83, 84]. IL-1β induces the production of chemoattractants CXCL1, CXCL2, and IL-17A by lung epithelial and Th17 cells that enhance the recruitment of macrophages and neutrophils [32, 85, 86]. Notably, since the pneumococcus is encapsulated and therefore resistant to phagocytosis and intracellular killing, this inflammatory response does not necessarily result in bacterial clearance and can worsen disease [87, 88].

When pneumococci kill host cells there is a release of alarmins and DAMPs including S100A8/9, HSP60, IL-1α, and IL-33, promoting the infiltration and activation of neutrophils and circulating monocytes which further the local immune response [32, 89, 90]. In professional antigen-presenting cells, alarmins have been shown to induce upregulation of surface costimulatory (CD80 and CD86) and MHC (class I and II) molecules, enhancement of antigen presentation to lymphocytes inducing adaptive immune responses, and the release of proinflammatory cytokines and chemokines, triggering a positive feedback loop on pro-inflammatory innate immune responses [90]. Additionally, alarmins can trigger mast cell and basophil activation and the release of histamine, prostaglandins, and leukotrienes thus, increasing vascular permeability and attracting neutrophils [90]. Bactericidal peptides and proteins released into the milieu include matrix metalloproteinases (MMPs); these zinc-dependent enzymes degrade extracellular matrix components, which then serve as alarmins, (e.g. hyaluronan) and thereby further increase the magnitude of the inflammatory response [58, 91–94].

Polymorphonuclear leukocytes (PMNs) are effectors of bacterial clearance and help to control the infection, even though they may be unable to phagocytize encapsulated pneumococci efficiently without opsonizing antibodies. They do this via the release of DNA and the formation of neutrophil extracellular traps (NETs). NETs trap and kill *Spn* with antimicrobial proteins associated with the DNA such as MMPs and serine proteases [31, 36, 59, 60]. Neutrophils also release agents that not only elicit inflammation but also cause tissue damage. These include reactive oxygen and nitrogen species, myeloperoxidase (MPO), and granule serine proteases such as cathepsin G, proteinase 3, and neutrophil elastase [32, 36, 57, 60]. The neutrophil influx necessary to fight off airway infection must be scaled appropriately to avoid excessive or prolonged inflammation [31, 94, 95]. Excessive inflammation sets the stage for the loss of alveolar-capillary barrier integrity, infiltration of additional neutrophils and monocytes, and initiation of a positive feed-forward pro-inflammatory loop.

During the early stages of pneumonia, the cytokine response to *Spn* is largely compartmentalized within the lung [96, 97]. However, as disease develops and becomes more severe, the inflammatory response in the airway becomes systemic with pro-inflammatory cytokines also becoming elevated in the blood of pneumonia patients [97–101]. Notably, a robust early response is vital, and patients who succumb to pneumonia have higher levels of cytokines and acute phase reactants in the serum versus the airway, indicating that failure to generate a robust local immune response at the site of initial infection contributes to systemic inflammation and overall disease severity [38, 96, 98]. TNF is one of the earliest and most important mediators of this local inflammatory response. TNF initiates inflammatory signaling cascades that induce a second wave of pro- and anti-inflammatory cytokines to advance the immune response [102]. TNF can also be damaging by causing increased neutrophil accumulation and activation, vascular dysfunction, and induction of necroptosis, a form of programmed cell death. [102–106]. Accordingly, levels of TNF in serum and the CNS correlate with overall disease severity and mortality [103, 107]. Patients who delayed going to the hospital > 48 h past the onset of symptoms had higher serum TNF levels, lower blood pressure (< 90 mmHg), and lower oxygen saturation levels at the time of admission; all of which are associated with poorer outcomes during infection [98]. Bacteremic patients have higher levels of TNF circulating in the serum compared to pneumonia alone [100, 108, 109]. Nearly all patients admitted to the hospital have a systemic inflammatory response, as measured by detectable IL-6 (98% in a study by Örtqvist et al.) and CRP; moreover, higher levels are associated with poorer outcomes [110, 111]. Compared to survivors, patients who died had higher levels of proinflammatory cytokines TNF, IL-6, IL-8, IL-10, and acute phase reactants (CRP) in serum [38, 96–98, 112]. Along such lines, polymorphisms in human genes involved in the inflammatory response to infection such as chemokines/cytokines (TNF, IL-6, IL-1 family, macrophage migration inhibitory factor, and IL-10), pattern recognitional molecules (TLRs and Mannose-binding lectin (MBL)), local host defense of the lung (surfactant protein), and coagulation cascades (plasminogen-activator-inhibitor (PAI)-1 and factor V) have been associated with susceptibility to pneumonia and outcomes including sepsis, IPD, and mortality [113–127]. This is due to an impaired ability to respond to the infection, but in other instances a far too exuberant response.

In summation, an inflammatory response is needed to fight off the potentially deadly pneumococcal infection. The immune system creates a highly inflammatory environment at the site of infection characterized by the recruitment of immune cells resulting in lung consolidation [128]. As the infection progresses, epithelial cell death and the subsequent loss of vascular integrity results in the leakage of pro-inflammatory bacterial products and cytokines into the systemic circulation that contributes to disease pathology. Scaling an appropriate immune response to avoid excessive inflammation is important to prevent damage from occurring, as an overzealous inflammatory response results in impaired oxygen exchange in the airway, damage contributing to organ injury, and increases the likelihood of poor outcomes, such as widespread organ failure and mortality [95, 129].

## Organ damage occurs during acute infection with lasting consequences

**Fig. 1:**
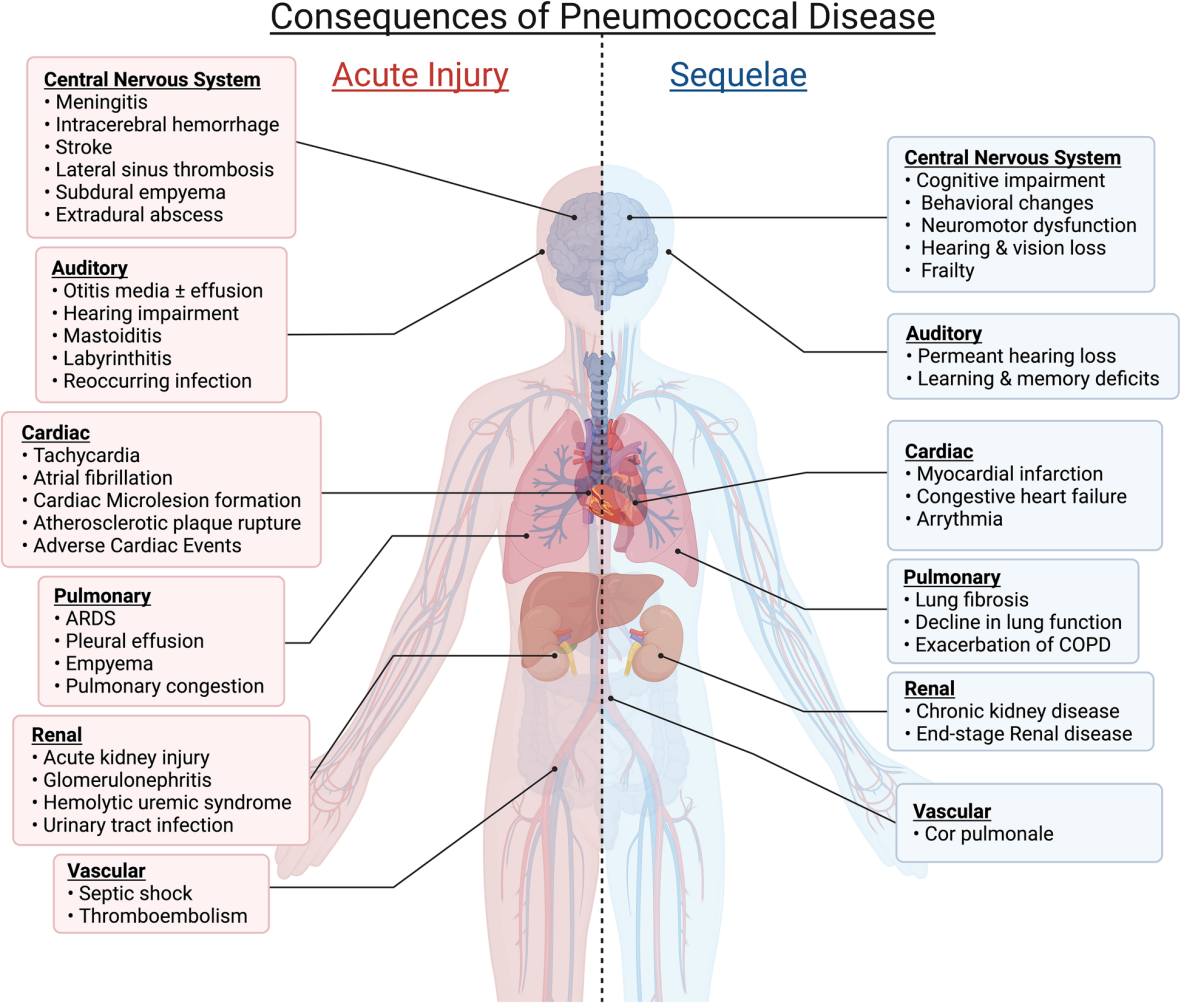
Consequences of pneumococcal disease during acute infection occur system-wide. Acute pneumococcal pneumonia and invasive disease can be life-threatening. It can also result in organ damage that results in debilitating long-term sequelae. These sequelae, in turn, contribute to loss of physiological resilience and early mortality

Sites of pneumococcal infection include the middle ear, lower airway, circulation, invaded organs, and the CNS. The consequences of severe infection at these locations can significantly and permanently alter human health (Fig. 1). Notably, work done in animal models shows unequivocally that bacterial-derived factors, as well as a dysregulated immune response, together contribute to this damage.

### Middle ear

#### Clinical aspects & pathophysiology

Otitis media is a common malady that affects the majority of children, with 80% having at least one episode by 10 years of age [11]. New infections often arise after viral infections or allergic reactions, as congestion obstructs the eustachian tube at its most narrow point, leading to the accumulation of middle ear secretions which create a niche for *Spn* to multiply within [130]. Children with otitis media often experience ear pain, otorrhea, difficulty hearing, and headache. Furthermore, ~60% of children develop otitis media with effusion (OME) that can persist for weeks to months following the resolution of the initial infection [130]. In the majority of otitis media cases, the tympanic membrane bulges, due to inflammation and fluid accumulation in the middle ear, but remains intact; however, spontaneous membrane perforation occurs in 4–30% of cases [9]. The majority of spontaneous perforations resolve within one month with favorable outcomes; however, 15% of cases become occluded, preventing drainage of pus from the middle ear and requiring medical intervention [9]. Children with perforations are twice as likely to have recurrent infections, which may be associated with permanent hearing loss [9]. Notably, populations with limited access to medical care, such as in indigenous communities or people in developing countries, are prone to developing complications of otitis media including labyrinthitis, mastoiditis, extradural abscess, subdural empyema, lateral sinus thrombosis, and meningitis [2, 10, 47, 130].

#### Bacterial factors

Swelling and death of inner and outer hair cells have been observed in the cochlea in experimental animal models as well as in human autopsies [23, 42, 131]. This damage was found to be dependent on the production of *Spn’s* pore-forming toxin, pneumolysin. Guinea pig cochleae perfused with pneumolysin had splayed hair bundle stereocilia and damaged, swollen hair cells that tore away from the supporting cells, ultimately resulting in hearing loss [23, 132]. Notably, chinchilla middle ears inoculated with purified cell wall of *Spn* exhibited the same histopathologic inflammation seen with live bacteria [123]. Otitis media is characterized as being highly neutrophilic with an abundance of neutrophil elastase, Cathepsin G, and lactoferrin, as well as the presence of NETs in middle ear effusions [133]. Myeloperoxidase generated by neutrophils to kill the bacterium has been shown to contribute to damage to the middle ear during otitis by generating hypohalous acids [30, 134].

#### Sequelae

Fluid present in the middle ear was shown to cause a hearing loss of 25 decibels (dB), similar to putting in earplugs, and the loss was 10 dB greater with bilateral disease [12]. Persistent effusion of the middle ear is often treated with tympanostomy tubes that provide relief by draining middle ear secretions to remove pressure and restore some hearing [11, 12]. In general, hearing substantially improves after one year although full recovery is a relatively slow process and children with a history of OME had defects in binaural processing 1 to 2 years past hearing recovery [135]. In addition to hearing loss, otitis media causes issues with vestibular balance and motor function that mostly return to normal following tympanostomy tube placement [47, 136, 137]. In children, recurrent episodes of otitis media may result in failure to reach cognitive developmental milestones, delayed or deficient speech development, and poorer school performance particularly in mathematics and reading [12, 13, 130].

### Lungs

#### Clinical aspects & pathophysiology

As pneumonia progresses, PMN infiltration occurs into bronchioles and adjacent alveoli with neutrophilic inflammation expanding into alveolar spaces in a manner that eventually obliterates the alveolar septa [138, 139]. Infection also results in the release of endogenous prostaglandins, which induce hypoxic pulmonary vasoconstriction of pulmonary artery blood flow into the consolidated lung resulting in an intrapulmonary shunt and increased oxygen consumption by the lung contributing to edema and arterial hypoxemia, referred to as acute lung injury. Additionally, carbon dioxide exchange is disrupted, increasing the respiratory rate, the minute ventilation, and the effort needed to breathe. Impaired surfactant activity reduces the dynamic compliance of the lungs, furthering the work needed to breathe effectively [140]. Typically, *S. pneumoniae* is associated with a lobar pattern that can be observed based on the pattern of opacity on chest radiographs. Lung congestion can be followed by the leakage of red blood cells (RBCs), neutrophils, and fibrin into the alveolar fluid, which changes the color of the lungs to dark red and for this reason is termed red hepatization [141]. Eventually, the RBCs associated with fibrinopurulent exudates break down transforming the lung from red to gray (i.e. gray heparinization) [141]. Resolution of infection is characterized by macrophage infiltration into alveolar space, clearing the exudates by scavenging dying neutrophils and other debris [138, 141].

During severe pneumonia, acute respiratory distress (ARDS) can develop. According to the American-European Consensus Conference, if the concentration of arterial oxygen in the blood divided by the inspired fraction of oxygen (i.e. a PaO2/FiO2) is less than 200 mm Hg a diagnosis of ARDS is made with the Berlin definition stratifying the severity of disease into 3 groups: mild (≤ 300 mm Hg), moderate (≤ 200 mm Hg), and severe (≤ 100 mm Hg) [28, 142, 143]. ARDS during severe pneumonia can be described as occurring in stages associated with direct damage to the lung parenchyma along with indirect damage incurred from systemic inflammation [28, 144]. In the first 1–6 days, there is interstitial and alveolar edema characterized by inflammation, thickening of the alveolar-capillary membrane, and the accumulation of neutrophils, macrophages, and red blood cells in the alveoli with evidence of both endothelial and epithelial injury [28, 144]. There is both denuding of the alveolar epithelium and the appearance of prominent hyaline membranes in the alveoli [144]. During the subacute phase (the next 7–14 days), some of the edema is reabsorbed and attempts at repair by the proliferation of alveolar epithelial type II cells along with infiltration of fibroblasts and increased collagen deposition [28, 144]. After 14 days, (the chronic phase), there is typically resolution of the acute neutrophilic infiltrate, but there are more mononuclear cells in the alveoli and more fibrosis and repair of the alveolar epithelial [28, 144]. The concentration of albumin, lactate dehydrogenase, IL-6, and neutrophil elastase in epithelial lining fluid of small airways is elevated in patients with ARDS [145, 146]. The edema and acute inflammation of ARDS is gradually resolved in many patients without fibrosis occurring [28]. The presence of ARDS increases the likelihood of non-pulmonary organ failure including cardiovascular failure, renal failure, abnormal liver function, and hematologic abnormalities such as anemia and thrombocytopenia [28, 142]. These are discussed in more detail below. In addition to ARDS, other pulmonary complications of pneumococcal pneumonia can occur including pleural effusion, empyema, multilobar infiltration, and abscesses or cavitations; having one or more of these complications is referred to as complicated pneumonia and is associated with higher rates of ICU admission, shock, a longer length of hospital stay, and treatment failure [147, 148].

#### Bacterial factors

Pneumolysin released from *Spn* causes lung damage through its cytolytic activity resulting in the impairment of pulmonary microvascular barrier function, damage-mediated influx of neutrophils, and severe pulmonary hypertension [28, 89, 106, 149–156]. Endothelial cells treated with pneumolysin had increased cell retraction and gap formation as well as detachment; pneumolysin-treated human alveolar epithelial cells also exhibited increased cell layer permeability [156]. Pneumolysin has been shown to induce respiratory epithelial cell necroptotic death as a result of ion dysregulation, causing the release of alarmins, and stimulating neutrophil elastase release after neutrophilic cell death, all of which contribute to lung injury [43, 57, 106, 157]. Pneumococci, through the conversion of pyruvate to acetyl phosphate by the metabolic enzyme streptococcus pyruvate oxidase (SpxB), produce profuse amounts of H_2_O_2_ [158]. This is known to induce oxidative stress, DNA damage, and apoptotic/necroptotic cell death in the lungs [158, 159]. Pneumolysin and H_2_O_2_ are especially potent together and have been implicated in the death of host cells [75, 77]. These molecules also have consequential negative effects even at concentrations below their cytotoxic threshold, for example slowing the beating of the cilia on epithelial cells [24, 25]. Notably, inhibiting macrophage apoptosis has been shown to decrease pneumococcal clearance from the lungs and promotes invasive pneumococcal disease as AM laden with *Spn* facilitate their own killing by undergoing apoptotic death [75, 77, 160].

#### Sequelae

In most instances, individuals with pneumonia recover fully. However, during severe cases, the physiological stress put on other organs and bacterial products or live bacteria in the circulation results in systemic organ damage. Patients with preexisting chronic obstructive pulmonary disease (COPD) were 42.3% more likely to experience exacerbations of disease if they experienced CAP [161]. Tachycardia is seen in many patients with pneumonia and can complicate pre-existing conditions to worsen cardiac function [162]. One feature of pneumonia, particularly in the elderly is loss of cognitive function and dementia. This is far more common and distinct from bacterial invasion of the CNS as discussed below. Many of these clinical features mirror those which are seen during sepsis.

Patients hospitalized for community-acquired pneumonia have an increased risk for long-term mortality that is nearly double that of patients hospitalized for all other combined causes [163–168]. Pneumococcal pneumonia results in a greater risk of death for at least 10 years following the acute bout of infection with 32.2% of survivors dying within 10 years [169]. Increased pneumococcal disease severity, as measured by the CURB-65 (confusion, urea > 7 mmol/L, respiratory rate ≥ 30/min, low blood pressure, and age ≥ 65 years) and/or the Pneumonia Severity Index (PSI) also known as the Pneumonia Patient Outcomes Research Team (PORT) risk score, is associated with greater long-term mortality [14, 170–173].

### Sepsis

*S. pneumoniae* is a primary cause of sepsis, and this has well-documented associations with poor outcomes such as mortality and the development of frailty. Sepsis, a life-threatening hyperinflammatory state, occurs due to the host being unable to clear *Spn* from the airway or bloodstream and the accompanying dysregulated host response to infection and organ failure [174–176]. Sepsis is a major contributor to the physiological stress and organ damage that ultimately leads to the sequelae following pneumococcal infection due to septic-associated dysfunction in the respiratory system, cardiovascular system, brain, liver, and kidneys. Notably, many excellent reviews on the pathophysiology of pneumococcal sepsis are available [175–181]. The features of sepsis overlap with those which occur during IPD, as in many instances these are often concomitant.

### Invasive pneumococcal disease

IPD refers to when the pneumococcus gains access to previously sterile sites including the bloodstream and CNS. Pneumococcal bacteremia occurs in roughly half of patients hospitalized with pneumonia and is associated with increased length of stay, the severity of infection, mortality, and risk of developing new morbidities [38, 182–185]. The mortality rate of patients with bacteremia is around 19% [184, 186]. Importantly once in the bloodstream, pneumococci are not restricted to the vasculature and are captured by / can invade filtering organs such as the liver, spleen, and kidneys. This puts the parenchyma of these organs at direct risk for damage if overwhelmed. The importance of the spleen in eliminating bacteria from the bloodstream is highlighted by the increased occurrence and magnitude of pneumococcal disease in asplenic individuals [187]. Likewise, splenectomized guinea pigs infected with *Spn* had increased bacterial titers in their blood, delayed clearance, and decreased survival [188]. It is now known that splenic CD169 + macrophages can be overcome by the bacteria and then serve as a “sanctuary” for replication of the bacteria and dissemination back into the bloodstream [189]. Additionally, there have been reported cases of splenic rupture during pneumococcal pneumonia [190]. Bacteria in the bloodstream can also invade the CNS and heart with devastating consequence. This is the result of direct translocation of pneumococci across vascular endothelial cells into organs. 

It is worth noting that organ damage incurred during IPD does not occur in isolation but instead accrues throughout multiple sites in the body simultaneously. This is due to the bacteria disseminating throughout the body. Inflammatory responses occurring concurrently in the lungs, vasculature, and within various organs exacerbate systemic inflammation and injury that can ultimately contribute to the development of sepsis. In addition to direct damage caused by the bacterium, biomechanical stress caused by alterations in circulatory volume and vascular resistance, inflammatory stress, increased metabolic demand, and other stressors associated with infection contribute to organ damage amassed during infection [191–193]. Collectively, this organ damage contributes to the sequelae of pneumococcal disease and long-term risk for mortality; survivors having increased risk of adverse events such as heart, renal, and neurological disease [49, 51, 194–196]. Accordingly, mortality rates among IPD survivors are greater than for individuals hospitalized for pneumonia without bacteremia [169]; one-third of patients who survived past 1-month end up dying within the next 10 years [169]. What is more, one 3-decade-long study from Ajayi et. Al, found that most patients that recovered from IPD died before their anticipated life expectancy with a mean potential life loss of 9.936 years [37]. In other studies the presence of bacteremia nearly doubled long-term mortality compared to blood culture-negative pneumonia [14, 37, 169]. Pneumococcal pneumonia in itself is a risk factor for the development of frailty with similar long-term mortality rates compared to chronic diseases, such as cardiovascular and cerebrovascular disease.

### Heart

#### Clinical aspects & pathophysiology

The correlation between pneumococcal pneumonia and acute heart failure has been recognized for over a century [41, 197, 198]. In the early twentieth century, patients with pneumococcal pneumonia were routinely prescribed Digitalis, a drug used to strengthen heart contractility [198, 199]. Hospitalization for pneumococcal pneumonia has been associated with new or worsened adverse cardiovascular events (CVEs) such as atrial fibrillation, heart failure, or myocardial infarction [162, 191, 192, 200]. In all, studies suggest that between a fifth to a third of patients admitted to the hospital for pneumonia develop cardiac complications [194]. Patients with preexisting cardiac conditions, such as heart failure, have an increased risk of disease exacerbations following pneumonia compared to a pneumonia-free cohort [161]. Over 90% of patients with pneumococcal disease who did experience cardiac complications had their first adverse cardiac event within the first week after admission to the hospital with > 50% occurring on the very same day [200]. Severe pneumonia episodes, in particular those with IPD, are positively correlated with increased short-term and long-term risk for adverse cardiac events. The presence of bacteremia increased the risk of adverse cardiac events. One of the best predictive measures of the severity of disease and in-hospital mortality was having higher troponin levels, a biomarker of cardiac damage, at the time of admission [162]. Supporting this notion, the 30-day mortality is higher in pneumonia patients with CVEs [200]. The increased incidence of CVEs during or following pneumococcal disease suggests that pneumococcal disease may exacerbate CVEs or even play a causal role.

Intense systemic inflammation present during pneumonia places stress on the heart which requires it to work harder due to decreased blood oxygen and lowered blood pressure that accompanies infection [162]. The increased levels of leukocytes, circulating inflammatory cytokines, CRP, coagulation factors, reactive oxygen species, and vasodilating molecules may increase the risk for thrombogenesis, procoagulant activity, and decrease cardiac contractility [201–203]. Myeloperoxidase produced from infiltrating neutrophils and monocytes has been implicated in cardiovascular disease as it can cause plaque rupture, damage artery walls, and activate MMPs causing further cardiovascular damage [204]. Infection promotes the development of thrombi by activating platelets, vascular constriction, dysregulation of the coagulation system, endothelial dysfunction, procoagulant changes in blood, inducing demand ischemia, and increases the risk for the instability and rupture of atherosclerotic plaque. Increased concentrations of troponins, BNP, and ANP have been associated with depression of left ventricular function and myocarditis. Increased pulmonary arterial pressure and levels of thrombin-antithrombin complexes, D-dimer, and plasminogen activator inhibitor along with decreased factor IX contributes to increased coagulation activation that is seen in almost 90% of patients hospitalized for pneumonia [200, 205, 206]. Alongside all of this, pneumococci also have a direct cytotoxic effect on cardiomyocytes.

#### Bacterial factors

**Fig. 2:**
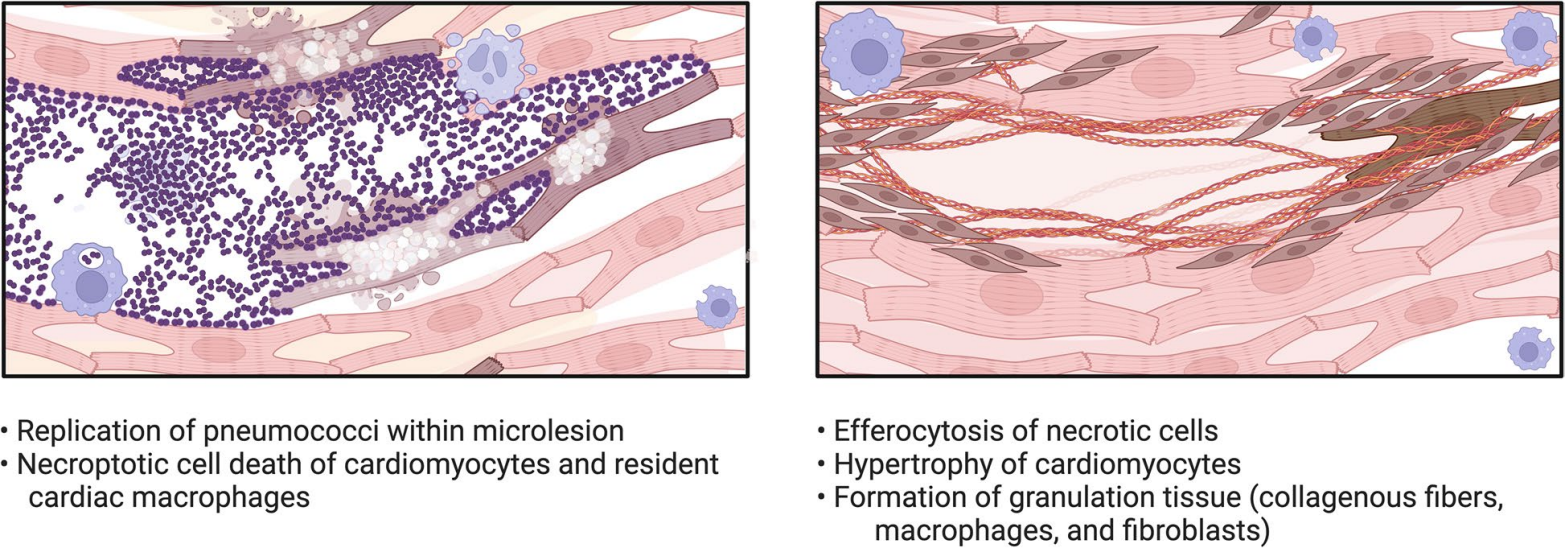
Cardiac microlesion formation and resolution. Microlesions form in the heart as a result of an individual pneumococcus adhering to the vasculature in the bloodstream and invading the myocardium by crossing vascular endothelial cells. Invaded pneumococci are capable of replicating to form a foci of infection (left panel). Infiltrating macrophages die of H_2_O_2_ and pneumolysin-mediated necroptosis preventing further infiltration of immune cells. Additionally, cardiomyocytes undergo necroptotic death leaving “holes” within the heart. The resolution of infection is characterized by the remodeling of cardiac tissue (right panel). This includes the infiltration of fibroblasts that deposit collagen to form long-lasting scar tissue. Microlesion morphology based on high-resolution images of cardiac sections from mice infected with *Streptococcus pneumoniae* strain TIGR4, shown 30 h post-infection, intraperitoneal challenge

Pneumococci-derived products including cell wall, H_2_O_2_, and pneumolysin are cardiotoxic and able to kill cardiomyocytes and suppress heart function. Pneumococcal cell wall components have been detected in the hearts of experimentally *Spn*-infected animals and shown to directly suppress cardiac contractility in a platelet-activating factor receptor-dependent manner [207]. Platelet-activating factor (PAF) activation in cardiac tissue is associated with long-lasting negative and arrhythmogenic effects on cardiomyocyte contractility [207–209]. Pneumolysin in the circulation is also able to cause cardiac damage, as pneumolysin-deficient *Spn* have less circulating troponins, biomarkers of cardiac injury, and cardiac damage when compared to pneumolysin-expressing *Spn* [210–212]. Cardiomyocytes exposed to pneumolysin experience an influx of Ca^2+^ into the cytoplasm through pneumolysin membrane-bound pores disrupting calcium signaling that is crucial for cardiac contractility in a dose-dependent manner [210]. Finally, work in non-human primates and mice has shown that *Spn* can translocate into the myocardium from the circulation in platelet-factor receptor and laminin receptor dependent fashion, establish a niche, and replicate to cause foci of infection called microlesions, serving to directly deliver cytotoxic products, including cell wall, pneumolysin, and H_2_O_2_ (Fig. 2) [45, 212, 213].

#### Sequelae

The transient risk of cardiovascular events occurring, including myocardial infarction or stroke, is 2–10 times greater following an acute respiratory infection [191–193, 200, 214, 215] and this is positively linked to its severity. Notably, admission to an ICU for pneumonia is associated with the greatest risk of new-onset heart failure, myocardial infarction, and cardiac-related death. Beyond acute infection, heightened risk for adverse cardiac events lingers for more than 5 years post-hospitalization [162, 192]. Work with primates and mice, suggests that upon successful antibiotic treatment, former microlesions are the site of extensive cardiac remodeling and collagen deposition [43, 157, 212]. Echocardiography of convalescent mice rescued with antimicrobials from IPD still had impaired ejection fractions and fractional shortening 3 months after successful intervention with antimicrobials [216]. Blocking of pneumolysin-induced cardiomyocyte death in experimentally infected mice rescued with antimicrobials reduced cardiac collagen deposition and reduced the extent of heart dysfunction in convalescence [216].

### Kidney

#### Clinical aspects & pathophysiology

Patients with chronic kidney disease are at increased risk of developing pneumococcal disease and vaccination is highly recommended in this group [217–219]. Independently, acute renal injury in the form of nephritis has been anecdotally associated with pneumococcal disease since 1872 [220, 221]. More recently, several studies have reported pneumococcal disease is damaging to the kidneys with consequences ranging from persistent proteinuria to end-stage renal disease [195]. Following hospitalization for pneumococcal pneumonia, one-third of patients experience acute kidney injury (AKI) [46, 222]. Those with AKI have increased hospital mortality, increased risk for adverse events including myocardial infarction and end-stage renal disease, and a nearly threefold greater rate of long-term mortality in those with moderate and severe injury [46, 222]. Among survivors, AKI can progress to chronic kidney disease as nephron loss leads to glomerular hypertrophy in the remaining nephrons [46, 223]. While *Spn* is not considered a typical agent of urinary tract infections (UTIs), it has been reported that children with chronic kidney disease have had urinary tract abnormalities associated with *Spn* infection and clinical UTI symptoms consistent with cystitis [224, 225]. Additionally, even in the absence of other disorders, *Spn* can be the causative agent of UTI, but due to the rarity of these causes more data is needed to evaluate the involvement of the pneumococci in the epidemiology and pathogenesis of UTIs [226].

Postulated mechanisms of renal injury during and following pneumococcal pneumonia include systemic hypotension and hypoxemia that contributes to peritubular hypoxia inducing fibrosis [195, 219, 227, 228]. Post-pneumococcal acute glomerulonephritis biopsy showed neutrophil and mononuclear cell infiltration into capillary loops with deposition of C3c and nephritis-associated plasmin receptors in the mesangial area and capillary loops [33]. Leukocyte-derived myeloperoxidase also contributes to glomerulonephritis [204]. Notably, pneumonia patients who develop acute kidney injury were older, had more comorbidities, and higher levels of inflammatory cytokines in the blood (IL-6, TNF, D-dimer) even in those without severe disease [222].

#### Bacterial factors

Although, case reports exist of *Spn* causing pyelonephritis and urosepsis [229, 230], there is overall a paucity of evidence that pneumococcal products or live *Spn* directly damage kidneys impairing function. Given what is known about the pneumococcus and since pneumococci are isolated from the kidneys of experimentally infected animals, it seems likely that a direct cytotoxic effect also contributes to the damage and therefore more research is warranted in this area. Along these lines, pneumolysin and bacterial neuraminidase have been implicated in damage [231–233]. Pneumococcal cell wall increasing levels of inflammation and prothrombotic molecules such as CRP, fibrinogen, and factor VII contribute to deteriorating renal function [195, 234]. Immune-complex-mediated acute glomerulonephritis is a rare complication of pneumococcal infection, and the pathogenic mechanism is hypothesized to involve the deposition of streptococcal antigen and formation of circulating immune complexes [33].

#### Sequelae.

*Spn*-associated hemolytic uremic syndrome (HUS) is an uncommon complication of pneumococcal pneumonia that develops 3–13 days after the onset of symptoms and is mostly observed in young children [235–237]. Pneumococcal HUS has a poor outlook with a mortality rate of 12.3% during the acute phase and ~ 10% of recovered patients going on to develop end-stage renal failure [236].

### Central nervous system

#### Clinical aspects & pathophysiology

Perhaps the most severe form of pneumococcal infection is meningitis, with case fatality rates varying from 20–37% in high-income countries and up to 51% in low-income countries where patients often face poor access to sufficient medical care. Cerebrovascular complications are very common during pneumococcal meningitis, with arterial stroke occurring in up to 30% of patients, cerebral venous thrombosis in 9%, and intracerebral hemorrhage in up to 9% [1, 238]. Pneumococci most likely gain access to the CNS via multiple sites including the blood-cerebrospinal fluid (CSF) barrier in the subarachnoid space, the blood–brain barrier (BBB) of the cerebral cortex, and the blood-CSF barrier in the choroid plexus [239, 240]; this is also the result of bacterial translocation across vascular endothelial cells and is dependent on pneumococci binding to platelet-activating factor receptor and laminin receptor. Additionally, children can develop meningitis, while having no obvious source of infection, in which bacteria gain access to the CNS directly from either the nasopharynx or middle ear. While CNS involvement most likely is preceded by bacteremia in adults, exceptions to this include those with craniofacial injury and hearing devices.

Much of the damage incurred during pneumococcal meningitis can be attributed to the inflammatory response to bacterial products [241]. CSF levels of inflammasome-associated cytokines, IL-1β and IL-18, correlate with systemic complications and worse clinical outcomes in patients [29, 242]. Blocking IL-1β reduces PM-induced cerebral inflammation and disease pathology [241, 243, 244]. MMPs released by neutrophils, neurons, and glial cells, among others, degrade the extracellular matrix contributing to decreased integrity of the subendothelial basal lamina of BBB, increased leukocyte invasion, inflammation, and neuronal injury [245]. Increased levels of myeloperoxidase produced by infiltrating leukocytes has been indicated in various neurodegenerative processes [204]. In experimental mouse models of pneumococcal meningitis, inhibition of MMP-2 and -9 decreased brain injury, lowered mortality, and prevented cognitive impairment [246, 247]. Remarkably, inflammation and leukocyte recruitment into the CNS occurred during bacteremia prior to pneumococci being detected in the brain [248]. Human histopathological studies from brains recovered from autopsies of pneumococcal meningitis patients have shown parenchymal damage secondary to intracranial pressure, cytotoxic and vasogenic edema, microhemorrhages, local leukocyte infiltration, abscess formation, fibrin thrombi, cortical necrosis, neuronal apoptosis in the hippocampal dentate gyrus, and hippocampal neuronal loss [48, 52]. Brain-diffuse ischemic axonal injury and Wallerian degeneration have been shown in brain autopsy and experimental pneumococcal meningitis [52, 249]. Brain structural damage can result from vasospasms and reduced blood flow eventually causing cortical necrosis and apoptotic cell death in the dentate gyrus [50]. Accordingly, blocking leukocyte invasion either by pharmaceutical inhibition of selectins or antibodies against endothelial intercellular adhesion molecules (ICAM1) or leukocyte integrin (Mac-1), attenuated the increased cerebral blood flow and intracranial pressure seen in early meningitis, inhibited leukocyte accumulation, brain edema, inflammation and BBB damage [29, 250, 251].

#### Bacterial factors

Pneumolysin, H_2_O_2_, and autolysin mediate brain cell mitochondrial damage and apoptosis of neurons through P38 MAPK activation, as well as intrinsic and extrinsic caspase-dependent programmed cell death [21, 22, 252–254]. Notably, the symptoms of meningitis can be replicated by injecting components of pneumolysin or *Spn*’s cell wall into the CNS; this is due to their cytotoxic properties and the inflammatory response generated in the CNS, respectively [255]. Meningeal inflammation significantly increased following antibiotic treatment attributed to bacterial lysis and release of cell wall [255]. However, by reducing the inflammatory response during antibiotic therapy using cyclooxygenase inhibitors some of the damage caused by host cells was mitigated [256]. Further, corticosteroid treatment has been shown to reduce CNS inflammation, severe morbidities, and case fatality rates [257].

#### Sequelae

Up to 30% of patients that survived meningitis have some type of neurological or neuro-behavioral sequelae [49]. These include seizures, hearing and vision loss, cognitive impairment, neuromotor disability, and memory or behavior changes [51]. In adult survivors of community-acquired pneumococcal meningitis from a Dutch nationwide prospective cohort study, 34% had persistent neurologic sequelae – most commonly hearing loss (27%). Neuro-physical evaluation revealed that patients who recovered from meningitis performed worse than controls with alertness, and cognitive flexibility with cognitive impairment seen in 14% of patients [22, 49, 51, 258]. Cognitive impairment occurred in all domains – most commonly cognitive speed (71%) but attention (60%) and memory (61%) were also affected. Survivors had lower quality of life scores, social functioning, and perceived health than those who had not experienced pneumococcal meningitis [49, 258]. Even healthy children and adults without apparent neurological deficits are at risk for long-term cognitive deficits following pneumococcal meningitis [259, 260]. Early identification and antibiotic/corticosteroid treatment is important to achieve a successful return to society [51].

## Frailty

Frailty is a state of vulnerability and loss of resilience to a variety of stressors that increase the risk of a variety of health-related problems that require hospitalization, including pneumonia [261, 262]. There seems to be a bidirectional relationship between pneumonia and cognitive function in which cognitive decline increases the risk for pneumonia while patients with pneumonia are at increased risk of subsequent dementia [260]. In older adults, hospitalization for pneumonia has been associated with a subsequent decline in physical and cognitive abilities along with the development of depressive symptoms [259]. Some patients that survived severe illness, such as ARDS, had persistent reduced physical quality of life 5 years after their illness, despite pulmonary function returning to normal. ICU- acquired weakness, or paresis, is common in survivors of critical illness and associated with worse outcomes, such as increased hospital mortality, long-term function, and quality of life outcomes [263–266]. Pneumonia is not only life-threatening but life-altering and can trigger a cycle of increased severity of frailty leading to accelerated aging, functional decline, and mortality.

## Premature mortality because of pneumococcal disease

While the majority of patients recover from respiratory symptoms within two to three weeks following infection, the general well-being of patients resolves more gradually [19, 267]. Many pneumonia survivors do not recover fully to their pre-infectious state after the cessation of antibiotics and still experience residual symptoms for months [19, 268–272]. Older patients and those with premorbid conditions take longer to recover and had a lower health-related quality of life as measured by physical functioning, and general and perceived health [19, 270]. However, it is important to note that while age is an important predictor of long-term mortality, pneumonia does also appear to cause excess long-term mortality in young populations as well [166, 273]. For example, 10% of patients were hospitalized within 30 days of their pneumonia diagnosis and nearly three-quarters of survivors were hospitalized again within a 5-year period for any cause [173, 192, 272]. The most common cause of death among pneumonia survivors was cardiovascular disease, followed by respiratory diseases and cancer [165–167, 173, 206, 274, 275]. Survivors are at increased risk for new-onset heart disease for up to 5 years post-hospitalization and strikingly 20% of survivors developed new or worsening congestive heart failure within 30 days of hospitalization [173, 191, 272].

One of the greatest predictors of long-term mortality following pneumococcal pneumonia is the presence and number of comorbid chronic conditions including cardiovascular disease, respiratory diseases (e.g. COPD), diabetes, kidney disease, liver disease, dementia, seizures, and cancer [19, 163, 185, 206, 273, 274, 276–281]. Several studies have found an association between the presence of co-morbidities and long-term mortality, with a larger number of co-morbidities predicting greater long-term mortality [163, 165, 167, 206, 273–285]. Additionally, a substantial body of evidence supports that advanced age is associated with increased long-term mortality after recovery from pneumonia [164–166, 168, 173, 273–276, 280, 281, 283, 284, 286]. Notably, racial disparity exists with black individuals having higher rates of both short-term and long-term mortality as well as an increased likelihood of developing additional morbidities such as heart failure, gastrointestinal bleeding, frailty, stroke, and myocardial infarction [287]. Potential reasons for these discrepancies in mortality include, genetics, socioeconomic factors, and the presence of additional co-morbidities which are disproportionately higher among African Americans, which together raise the risk for severe infection [279, 288–293]. Evidence specific to older adults suggests that older adults exhibit excessive pro-inflammatory cytokine levels after acute pneumonia that are associated with morbidity and mortality [290, 294–296]. In accordance with this, higher systemic levels of pro-inflammatory cytokines, such as IL-6 and TNF, at discharge can predict both short and long-term mortality after hospitalization for pneumonia [110, 146, 294, 295, 297, 298]. Male sex appears to accentuate this age-related dysfunction in the resolution of inflammation [298–302]. In addition to inflammatory cytokines, several studies have looked into various biomarkers to attempt to predict short and long-term outcomes, showing that high serum glucose, blood urea nitrogen/albumin ratio, creatinine, pro-atrial natriuretic peptide, pro-endothelin-1,pro-vasopressin, pro-adrenomedullin, CRP, troponins, hemostasis markers (such as D-Dimer, thrombin-antithrombin complexes, factor IX, antithrombin, and plasminogen activator inhibitor-1), and white blood cell count, as well as low serum albumin and alanine aminotransferase all had a high predictive power for short and long term mortality, with pro-adrenomedullin consistently being one of the best indicators of outcomes [110, 167, 182, 205, 206, 273, 284, 303–306]. The clinical power in utilizing biomarkers, such as pro-adrenomedullin, along with pneumonia severity indexes should be used to identify and guide treatment in patients at high risk for the development of new morbidities or mortality so they can be carefully monitored to attempt to reduce long-term consequences [182].

## Treatment and prevention

Currently, the most effective strategy for clinicians to address pneumococcal infections is prompt access to care, accurate diagnosis, rapid antibiotic delivery, and disease prevention by implementing widespread vaccination against the pneumococcus [307]. Many groups have published guidelines for the management of pneumococcal disease, including the American Thoracic Society (ATS), Infectious Diseases Society of America (IDSA), and the British Thoracic Society (BTS) that indicates patients should be treated with an agent that specifically targets pneumococcus, recommending monotherapy with β-lactams for uncomplicated outpatients and a combination of β-lactam and macrolide therapy for severe cases [308–315]. Early administration of antimicrobials is associated with a better prognosis and the failure to use antibiotics concordant with IDSA and ATS guidelines has been associated with an increased risk of in-hospital mortality and more than a five-fold increase in 30-day mortality [316–325].

The Advisory Committee on Immunization Practices (ACIP) recommended adults ≥ 65 years and adults 19–64 with underlying medical conditions or other risk factors be vaccinated with a polysaccharide-conjugate vaccine, either a combination of PCV15 followed by PPSV23, the pneumococcal polysaccharide vaccine, or PCV20 alone [326]. Annual vaccination against seasonal influenza viruses is recommended in all patients as influenza infection predisposes individuals to pneumococcal infection [326–328]. Additionally, individuals with chronic kidney disease are recommended to receive pneumococcal conjugate vaccines as this has been shown to reduce vaccine-type IPD by 75% in these individuals as well as lower overall mortality especially when a pneumococcal vaccine is used in combination with an annual influenza vaccine [217, 218, 329]. This is crucial as patients with end-stage renal disease are between 10 and 16-fold more likely to die from pneumonia than the general population [217, 227, 228, 329]. Vaccination against *Spn* has also been effective in reducing meningitis however serotype replacement has led to an emergence of non-vaccine serotypes that have reduced the overall effect of vaccination on pneumococcal meningitis incidence in Europe and North America [330, 331]. The Global Vaccine Action Plan has committed to extending the availability of pneumococcal vaccines to low and lower-middle-income countries in an effort to relieve some of the burden of pneumococcal disease [332]. Following the introduction of PCV10 in Brazil, there was a 10% decline in pneumonia mortality in children, and similar large benefits are expected when PCVs are introduced in other low-income settings [333]. Currently, many countries routinely vaccinate adults but there is controversy in policies such as whether the use of PCV and/or PPV is clinically and cost-effective, what age groups should be vaccinated, and whether repeat dosing could prove beneficial [334–338].

There is controversy that exists on the efficacy of pneumococcal vaccination in reducing overall long-term mortality and the consequences of pneumococcal disease. However, research has shown pneumococcal vaccination to be effective at preventing pneumonia and IPD in older adults who are at higher risk [339–346]. With an aging population that is expected to double by the year 2050, it is becoming increasingly important to extend vaccination for the promotion of healthy aging [347, 348]. Considering the drastic long-term effects that pneumonia can have, pneumococcal vaccination should be implemented to reduce pneumococcal disease burden as research has shown that even slight clinical effectiveness is highly cost-effective. Indeed, vaccination in the elderly has been associated with lower hospitalization rates for pneumonia, mortality, and medical cost [349]. PPV is effective at preventing IPD, even in immunocompromised patients [335]. Routine infant immunization with PCV has reduced all-cause pneumonia hospital admissions, cases of acute otitis media, IPD, and mortality in children under 5 [350–353]. While research investigating the effect of vaccination on the long-term consequences of pneumonia is scarce, considerable evidence does support the beneficial effects of the pneumococcal vaccination, and that wider usage of vaccination should be implemented.

Pneumonia is currently considered the 9^th^ leading cause of death, but this estimate only takes into account short-term mortality of up to 30 days and likely underestimates the overall morbidity and mortality that results from the pneumococcus [354]. Evidence supports that *Spn* contributes to the new development or worsening life-threatening morbidities and long-term mortality in coming years. Further research is needed to understand the long-term sequelae of pneumococcal disease to come up with better preventatives and treatments, so this Captain of death no longer lingers.

## Data Availability

Not applicable.

## Abbreviations

*Spn*: *Streptococcus pneumoniae*
CAP: Community-acquired pneumonia
CNS: Central nervous system
AMs: Alveolar macrophages
MMPs: Matrix metalloproteinases
CRP: C-reactive protein
PRRs: Pathogen recognition receptors
TLRs: Toll-like receptors
NLRs: NOD-like receptors
PAMPs: Pathogen-associated molecular patterns
DAMPs: Damage-associated molecular patterns
MAPKs: Mitogen-activated protein kinases
NF-κB: Nuclear factor kappa B
TNF: Tumor necrosis factor
IL: Interleukin
PMNs: Polymorphonuclear leukocytes
NETs: Neutrophil extracellular traps
MPO: Myeloperoxidase
OME: Otitis media with effusion
dB: Decibels
ARDS: Acute respiratory distress syndrome
RBCs: Red blood cells
COPD: Chronic obstructive pulmonary disease
IPD: Invasive pneumococcal disease
CVEs: Cardiovascular events
AKI: Acute kidney injury
HUS: Hemolytic uremic syndrome
UTIs: Urinary tract infections
CSF: Cerebrospinal fluid
BBB: Blood–brain barrier
